# Integrated Analysis of miRNAome and Transcriptome Identify Regulators of Elm Seed Aging

**DOI:** 10.3390/plants12081719

**Published:** 2023-04-20

**Authors:** Tiantian Ye, Xu Huang, Tianxiao Ma, Ying Li, Xiaofeng Wang, Hai Lu, Hua Xue

**Affiliations:** State Key Laboratory of Tree Genetics and Breeding, National Engineering Research Center of Tree Breeding and Ecological Remediation, College of Biological Sciences and Biotechnology, Beijing Forestry University, 35 Tsinghua East Road, Beijing 100083, China; yetiantian1994@bjfu.edu.cn (T.Y.);

**Keywords:** seed aging, seed vigor, miRNA, miRNAome, degradome, transcriptome, *Ulmus pumila* L.

## Abstract

After maturity, seed vigor irreversibly decreases. Understanding the underlying mechanism is important to germplasm preservation. MicroRNAs (miRNAs) play vital regulatory roles in plants. However, little is known about how miRNAs regulate seed aging. Here, elm (*Ulmus pumila* L.) seeds of three aging stages were subjected to a multi-omics analysis including transcriptome, small RNAome and degradome, to find regulators of seed aging. In the small RNAome, 119 miRNAs were identified, including 111 conservative miRNAs and eight novel miRNAs specific to elm seeds, named upu-miRn1-8. A total of 4900 differentially expressed genes, 22 differentially expressed miRNAs, and 528 miRNA-target pairs were identified during seed ageing. The target genes were mainly involved in the processing of proteins in the endoplasmic reticulum, metabolism, plant hormone signal transduction, and spliceosome. The expression of several DEGs and miRNAs were verified by qRT-PCR. The degradome data showed the exact degradation sites of upu-miR399a on *ABCG25*, and upu-miR414a on *GIF1*, etc. The dual-luciferase assay verified the negative regulation of upu-miR399a on *ABCG25* and upu-miR414a on *GIF1* in tobacco leaves. This study outlined the regulation network of mRNA, miRNA and miRNA-target genes during seed aging, which is helpful in integrating the regulation mechanisms of seed vigor at the transcriptional and post-transcriptional levels.

## 1. Introduction

After maturity, the irreversible decline in seed vigor during storage is called seed deterioration or aging, which causes a serious problem in germplasm conservation [1]. Although low temperature storage is a relatively ideal method of seed storage, deterioration will still occur with the extension of storage time. As seed vigor decreased, programmed cell death (PCD) occurred, and the structure of mitochondria, the cell membrane, and nuclei was progressively destroyed. At the molecular level, DNA fragmentation, protein degradation and lipid peroxidation [2,3] are hallmarks of cell death. The levels of reactive oxygen species (ROS) [4,5], nitric oxide (NO) [6,7] and other signal molecules changed significantly. With the tremendous changes in the whole cell, the miRNA may play an important regulatory role.

MicroRNA (miRNA) is a class of small non-coding ribonucleic acid (RNA) [8], which is produced by precursor miRNAs (pre-miRNAs) and integrated with RNA-induced silencing complexes (RISC). It could regulate gene expression by degrading or silencing target genes [9] in order to regulate various life activities such as plant growth, development, and stress response [10]. During seed development, germination and dormancy, miRNAs and their target genes form a huge regulatory network. Wei et al. [11] combined the miRNAome with the degradome to analyze Brassica seeds during development, and obtained a batch of differentially expressed miRNAs and target genes regulating seed development and lipid synthesis. In banana ripening, the target genes of 82 differentially expressed miRNAs were found to encode transcription factors and other functional proteins, including SPL, APETALA2, EIN3, E3 ubiquitin ligase, β-galactosidase, and β-glucosidase [12]. Xu et al. [13] identified two TIR1-like genes, *CsTIR1* and *CsAFB2,* in cucumber, and found that transgenic tomatoes showed a phenotype of smaller seeds with reduced numbers and decreased germination percentage, which were negatively regulated by miR393. In a study of the dormancy mechanism of Chinese fir seeds, miRNA-mediated mRNA degradation is also an important mechanism to relieve primary dormancy [14].

Seeds are major materials for plant reproduction, and seed vigor is essential for agricultural and forestry production. In recent years, miRNAs related to seed vigor and longevity has been identified in maize, rice, and other plants. Gong et al. [15] conducted a comprehensive analysis of miRNAs in different artificial aging treatments of corn, and found that up-regulated Zma-miR319a-3p_R+1 is the highest expression miRNA when corn seed vigor declined, while bdi-miR159-3p is down-regulated. Compared with untreated Oryza seeds, 11 miRNAs were differentially expressed after artificial aging, among which miR164 was up-regulated and miR168 was down-regulated [16]. In miR164c-silenced line MIM164c and the miR164c overexpression line OE164c of rice, miRNA1846, and miRNA531a-c took part in the oxidative phosphorylation pathway, and miR5075 was involved in the oxidoreductase pathway [17]. Twenty-seven miRNAs were found to be differentially expressed in the control check (CK) and aged sweet corn seeds. Among them, PC-5p-213179_17 targeted peroxidase superfamily protein, which might affect seed vigor by regulating the redox process and responding to oxidative stress [15]. However, the role of miRNA in the ageing of woody seeds was less investigated and largely unknown.

Control deterioration treatment (CDT) is a classical method to study seed ageing. In our previous study, elm seeds could lose their vigor under CDT within a week, making it an ideal model for the ageing study of woody seeds. There are numerous sequences that code for microRNAs, and these are related to the regulation of the gene expression of practically any trait, including seed deterioration. Here, we hypothesized that miRNAs played crucial roles during the ageing of elm seeds. The specific objectives of this study were to identify the key miRNAs and genes involved in elm seed aging and to explore their relationship in seed vigor regulation. To this end, elm seeds of different aging stages by CDT were subjected to RNA-seq, multi-omics analysis, and the expression confirmation of individual miRNAs/target genes. Our data provide significant information to determine the gene regulatory network in the seed ageing, and is of great significance for maintaining seed vigor and improving storage performance.

## 2. Results

### 2.1. Small RNA Profiles and miRNA Identification in Aged Elm Seeds

**Figure 1:**
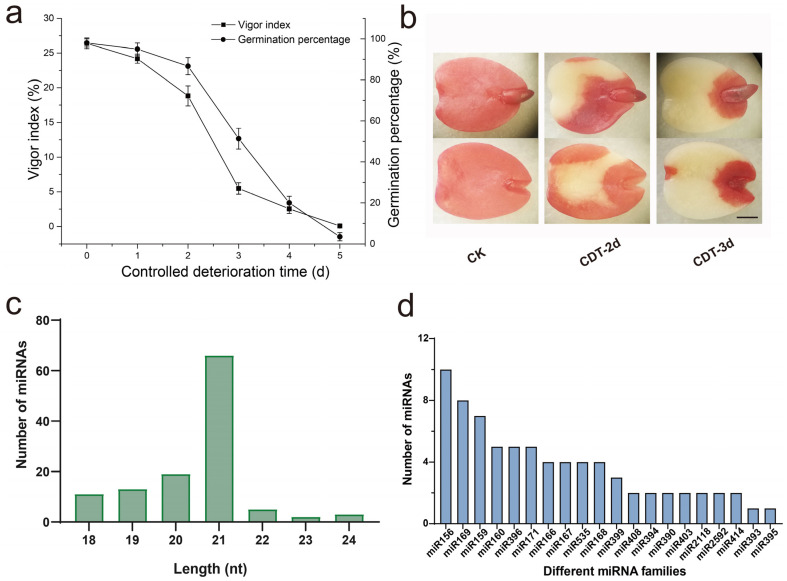
Small RNA profiles and miRNAs identification in aged elm seeds. (**a**) Effects of controlled deterioration treatment (CDT) on germination and the vigor index of elm seeds. (**b**) TTC staining of elm seeds after CDT. The TTC stained cotyledons were observed by micrographs. Control check seeds (CK), seeds aged for two days (CDT−2d), and for three days (CDT−3d) were used for multi-omic analysis. Scale bar = 1 mm. (**c**) Length distribution of the miRNA sequences in the miRNAome. The abscissa represents the length of the mature miRNAs, and the ordinate represents the number of miRNAs contained in each length of the miRNA. Nt, nucleotide. (**d**) The count of the top 20 miRNA family. The abscissa represents names of different miRNA families, and the ordinate represents the number of members contained in each miRNA family.

During aging of elm seeds, the germination percentage decreased to 80% after two days of CDT, and further decreased to 50% after three days of CDT (Figure 1a). TTC staining was used to detect the cell viability of aged elm seeds. The whole seeds without CDT were dyed dark red. With the extension of CDT, the successful dyeing area by TTC was gradually reduced, indicating that the seed vigor of elm was continuously decreasing during CDT (Figure 1b). To investigate the role of miRNA in the aging process of elm seeds, we constructed three small RNA libraries using sRNAs obtained from the seeds of CK, CDT-2d, and CDT-3d, respectively. Through high-throughput sequencing, 889, 675 (CK), 526,319 (CDT-2d) and 612,420 (CDT-3d) clean reads were obtained from these three libraries. Among them, 5,014,200 (CK), 5,137,091 (CDT-2d) and 4,042,332 (CDT-3d) valid reads were obtained for subsequent analysis after the further removal of rRNA, snoRNA, snRNA, tRNA, and other RNA (Table S1). The value of Q30 was higher than 85%, indicating that our small RNA sequencing quality was good. The displayed length of miRNA sequences in the three libraries was mainly enriched at 18–24 nt. The small RNA from each library had a similar distribution pattern, in which the 21-nt small RNA was the most distributed (Figure 1c).

MiRNAs are highly conservative and play important roles in various biological processes in plants. As the genome sequence for elm is not yet available, the transcriptome data was used as a reference for further analysis. The miRNAs of elm seeds were aligned with known plant miRNAs in the miRBase database (V22.1), and the same sequences with other species were identified as the conservative miRNAs of elm seeds. The inverted repeats and stem-loop structures were identified according to the transcriptome sequences of elm seeds. A total of 111 conservative miRNAs of elm seeds were identified, belonging to 30 families. Most conservative miRNA families are composed of several different mature sequences. The conservative miRNAs with the most members were miR156, including ten members, followed by the miR169 and miR159 families, with over six members. The member numbers of the top 20 miRNA families are shown in Figure 1d. To identify the miRNAs involved in the aging of elm seeds, the normalized expressions of miRNAs in each sample were compared. It showed that the expression levels of different miRNA families showed different scales of change. The top three abundance mature miRNA sequences were upu-miR156f, upu-miR156l, and upu-miR166a, with an abundance ranging from 27,219.33 to 169,591.27 (Table S2).

Unannotated sRNA reads are used to predict novel miRNAs. We searched for putative precursors from our elm mRNA transcriptome database to further characterize the sequenced miRNA candidates. Each miRNA precursor has a higher minimum folding free energy index (MFEI). Based on the criteria for plant miRNA annotations [18], a characteristic stem-loop precursor is a prerequisite for new miRNA annotation. A total of eight specific miRNA sequences were identified as candidate miRNAs, enriching the number of miRNAs in elm seeds. The specific miRNA in elm seeds is named in the form of ‘upu-miR + n + number’, as upu-miRn1 to upu-miRn8 (Table S3). All of the putative precursors of novel miRNAs had regular stem-loop structures, and the predicted hairpin structures were analyzed by the mfold software v3.1, producing mature miRNAs from 21 to 24 nt. The MFEI of novel miRNAs is between 0.9 and 3.1. Compared with the conservative miRNAs, the abundance of novel miRNAs was significantly lower. Among them, upu-miRn5 and upu-miRn6 showed a higher abundance. These miRNAs are first found in elm seeds, which may play special roles in them.

### 2.2. Dynamic Expression of miRNA during the Aging of Elm Seeds

**Figure 2:**
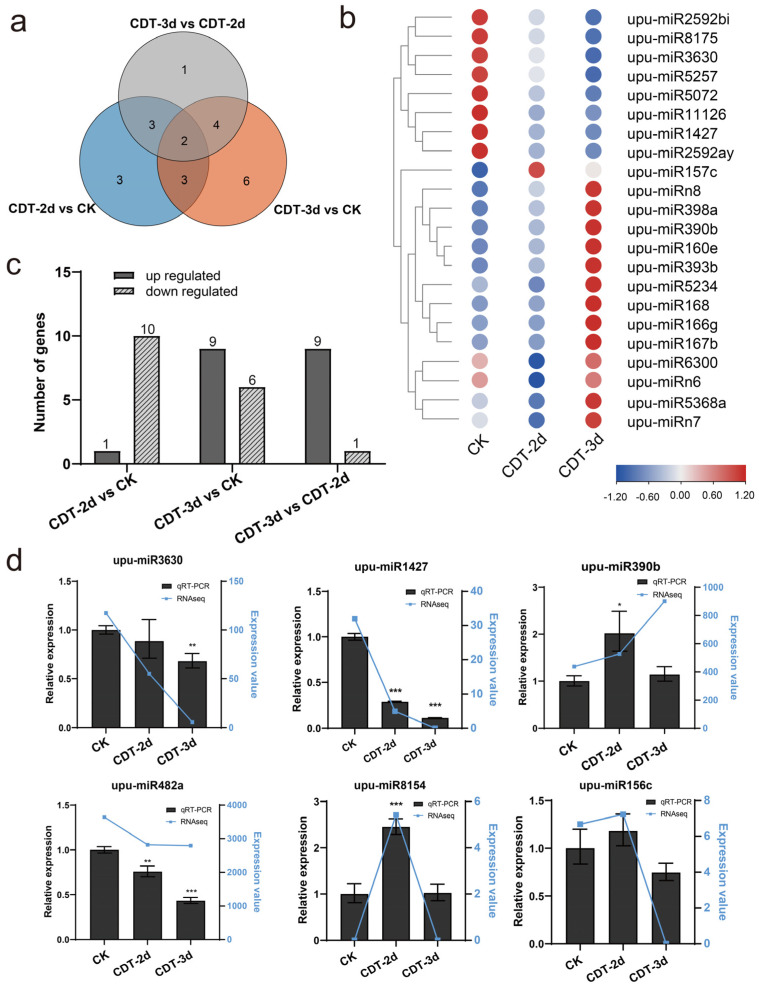
Differentially expressed miRNAs (DEMs) during seed aging in miRNAome and expression verification by qRT−PCR. CK, control check; CDT−2d, seeds aged for two days; CDT−3d, seeds aged for three days. (**a**) Venn diagrams show the number of common and specific miRNAs in comparison with the three libraries. The Venn diagram was plotted using TBtools v1.108. (**b**) Expression patterns of the DEMs based on deep−sequencing datasets. The blue−red bar at the bottom right of the heat map indicates the relative expression intensities. The darker the red color, the higher the expression, while the darker the blue color, the lower the expression. Expression pattern diagrams of miRNAs were plotted using TBtools v1.108. (**c**) The bar chart shows the number of up-regulated and down-regulated DEMs in comparison with the three libraries, with FDR ≤ 0.05, |log_2_FC| ≥ 1 as the threshold. The bar graphs were plotted with GraphPad Prism v8.0.1. (**d**) The comparison of qRT−PCR and the sequencing results of six miRNAs. A total of 5.8s rRNA of elm was used as an internal reference for miRNA. All reactions were repeated three times. Error bars indicate the standard deviation of three replicates. *, *p* < 0.05; **, *p* < 0.01; ***, and *p* < 0.001. The expression data by miRNAome sequencing are shown by the broken blue line for the sake of comparison.

Elm seeds with different aging stages exhibited different miRNA expression profiles. To identify miRNAs involved in seed aging, we profiled miRNAs with alterations in expression levels during the aging of elm seeds. The results showed that the CDT changed the abundance of some miRNAs in elm seeds. With the threshold of |log_2_FC| ≥ 1 and *p* value < 0.05, the DEMs were screened. The expression of twenty-two miRNA was changed, including nineteen conservative miRNAs and three novel miRNAs (Figure 2a,b). Compared with CK, ten miRNAs were down-regulated and one miRNA was up-regulated in CDT-2d, while nine miRNAs were up-regulated and the expression of six miRNAs decreased in CDT-3d. Compared with CDT-2d, nine miRNAs in the CDT-3d sample were up-regulated, while one miRNA was down-regulated (Figure 2c). The expression patterns of the DEMs during seed ageing were then drawn based on deep-sequencing datasets (Figure 2b). It was found that eight miRNAs including upu-miR2529bi, upu-miR3630, and upu-miR1427 showed a down-regulation trend during aging, while upu-miR398a, upu-miR390b, as well as the novel miRNAs upu-miRn8 and upu-miRn7 were up-regulated after CDT. These DEMs may perform certain functions during seed aging.

To verify the reliability of small RNA sequencing data, the expression of six miRNAs: three from DEMs and three others, were detected by qRT-PCR. The sequencing results are shown by the broken blue line for the sake of comparison. We confirmed that the levels of upu-miR3630, upu-miR1427, and upu-miR482a were down-regulated during seed aging, while the expressions of miR390b, upu-miR8154, and upu-miR156c were up-regulated in CDT-2d, and then down-regulated in CDT-3d. According to the results verified by qRT-PCR, the expression patterns of the majority miRNAs were consistent with those of small RNA sequencing profiles, except for miR390b in CDT-3d (Figure 2d). The reliable miRNA sequencing results will provide a foundation for further study.

### 2.3. Dynamic Transcriptome Changes of Genes during Elm Seed Aging

**Figure 3:**
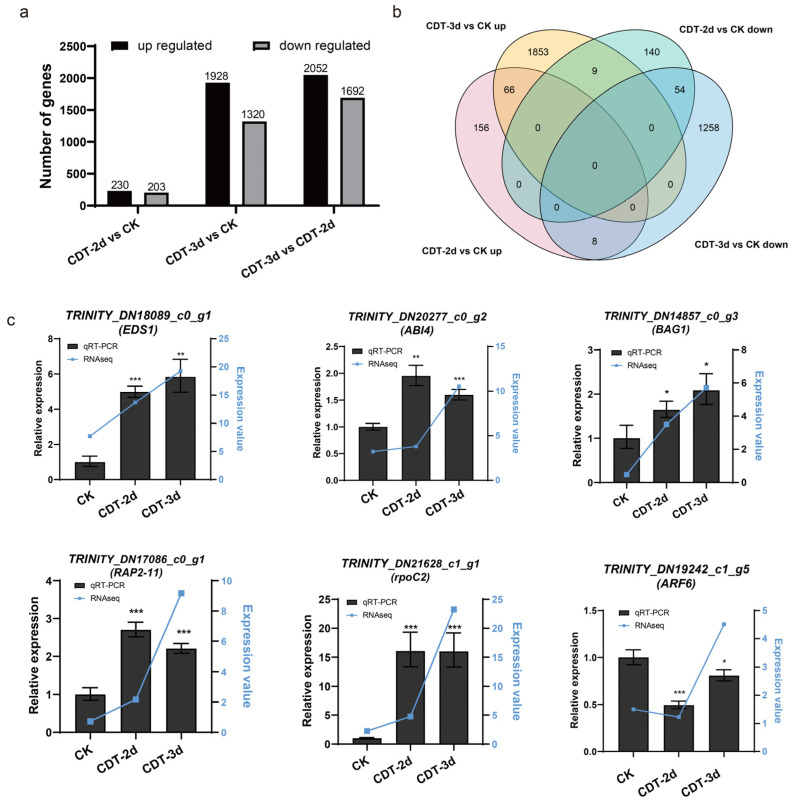
Differentially expressed genes (DEGs) during seed aging in transcriptome and expression verification by qRT−PCR. CK, control check; CDT−2d, seeds aged for two days; CDT−3d, seeds aged for three days. (**a**) The bar chart shows the number of up−regulated and down−regulated DEGs in comparison to the three libraries, with FDR ≤ 0.05, and|log_2_FC| ≥ 1 as the threshold. (**b**) Venn diagrams show the number of genes up and down−regulated during the aging of elm seeds. (**c**) The comparison of qRT−PCR and sequencing results of six genes. The actin of elm was used as an internal reference. All reactions were repeated three times. Error bars indicate the standard deviation of three replicates. *, *p* < 0.05; **, *p* < 0.01; ***, *p* < 0.001. The expression data by transcriptome sequencing are shown by the broken blue line for the sake of comparison.

Transcriptome data could reveal the regulation network on the transcriptional level, and provide expression profile of genes targeted by miRNAs. To examine changes in the transcriptome during the aging of elm seeds, we performed a global analysis of mRNA expression in seeds of non-aged control check (CK), CDT-2d, and CDT-3d. We constructed three transcriptome libraries and obtained 45,716,170 (CK), 47,292,674 (CDT-2d), and 52,641,304 (CDT-3d) raw reads. The raw data were pre-processed to remove adaptor sequences, duplicate reads, and low-quality reads (Table S1). The value of Q30 was more than 95%, indicating that our transcriptome data was qualified for further analysis. Through further analysis, with FDR ≤ 0.05 and |log_2_FC| ≥ 1 as the threshold, a total of 4900 DEGs were found (Table S4). There are 433 DEGs in CDT-2d, including 230 up-regulated genes and 203 down-regulated genes when compared to CK. In the CDT-3d sample, 3248 DEGs were found when compared to CK, while 3744 DEGs were found when compared to CDT-2d (Figure 3a). With the decrease in seed vigor, the number of DEGs dramatically increased (Figure 3a and Figure S1a), indicating that there were more genes and regulatory mechanisms involved as seed vigor further declined. During seed aging, 66 genes were continuously up-regulated and 54 genes were continuously down-regulated, including *MPK12*, *HSP70*, *DNAJ*, *SF3B1*, and *NAD*, suggesting that these 120 genes might play an essential role in seed aging (Figure 3b, Table S4).

To study the biological functions of these DEGs, we further carried out Gene Ontology (GO) and a Kyoto Encyclopedia of Genes and Genomes (KEGG) analysis on these DEGs. In GO analysis, the top 10 GO terms with the lowest *p* value were enriched for mapping. The abscissa is the GO entry, and the ordinate is the −log_10_ *p* value of the enrichment analysis. Results of the GO enrichment showed that the biological processes were mainly related to the response to chitin, flavonoid glucuronidation, and the abscisic acid-activated signaling pathway during seed aging. The cellular components were mainly enriched in the endosome, and the activity of the glucosyltransferase was associated with seed ageing (Figure S1b). The enrichment results of KEGG showed that up-regulated genes were mainly enriched in protein processing in the endoplasmic reticulum, plant hormone signal transduction, and MAPK signaling pathway (Figure S1c). During seed aging, down-regulated genes were mainly enriched in oxidative phosphorylation (Figure S1d). The enrichment results of differentially expressed genes at different aging stages are shown in Table S4.

The expression of six genes *EDS1* (*protein EDS1L like*), *ABI4* (*ethylene-responsive transcription factor ABI4-like*), *BAG1* (*BAG family molecular chaperone regulator 1*), *RAP2-11* (*AP2/ERF domain containing protein*), *rpoC2* (*putative DNA-directed RNA polymerase*), and *ARF6* (*auxin response factor 6*) were detected by qRT-PCR to verify the reliability of the transcriptome data. We confirmed that the expression patterns of the six genes were similar to those in the transcriptome (Figure 3c), indicating that the transcriptome data was reliable for use for further analysis. Overall, these results describe the dynamic expression of genes in elm seeds during aging.

### 2.4. Identification of miRNA Target Genes by Degradome Sequencing

**Figure 4:**
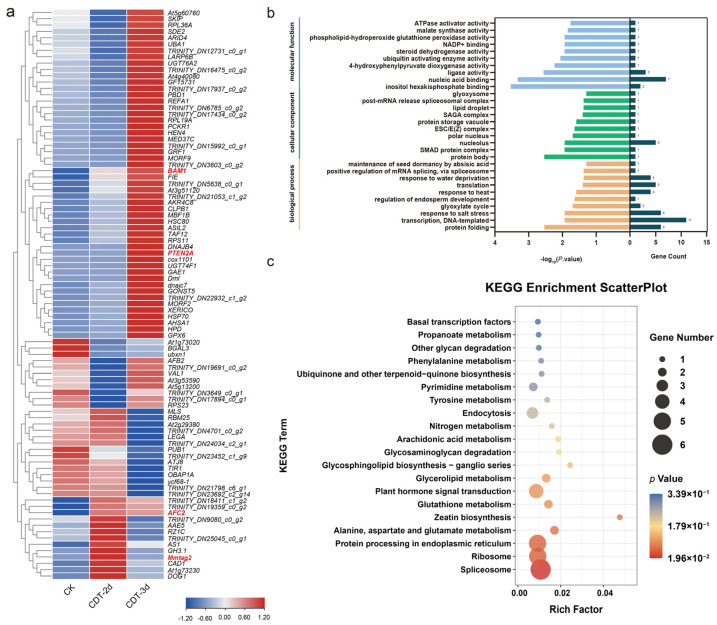
Overview of identified target genes of DEMs by degradome sequencing. (**a**) Expression profile of target genes of DEMs. CK, control check; CDT−2d, seeds aged for two days; CDT−3d, seeds aged for three days. The genes further analyzed by experimental approaches are highlighted in red. The blue−red bar at the bottom right of the heat map indicates the relative expression intensities. The darker the red color, the higher the expression, while the darker the blue color, the lower the expression. The expression pattern diagrams of miRNAs were plotted using TBtools v1.108. (**b**) The top 10 GO terms with the lowest *p* value were enriched for mapping. The ordinate is the GO entry, the left abscissa is the −log_10_ *p* value of the enrichment analysis, and the right abscissa is the gene number. (**c**) KEGG enrichment analysis for target genes of DEMs.

MiRNAs negatively regulate their targets by inhibiting or degrading them. In order to determine whether some changes in the transcriptome were related to the miRNA during seed aging, Split-site prediction software GSTAr v1.0 [19,20] was used to predict the target genes of miRNA in CK, CDT-2d, and CDT-3d. A total of 16,737 target genes of 119miRNA were identified, including 1231 targets of eight novel miRNA (Table S5). Furthermore, we performed degradome sequencing to predict the target genes of miRNA in mixed samples of CK, CDT-2d, and CDT-3d. The target gene corresponding to the predicted miRNA was combinatively analyzed with the mRNA in the generated degradation group density file, and the common mRNA was identified to be the target gene of miRNA. A total of 8,524,284 raw reads (64.12% mapped) were obtained from the degradome, including 2,811,946 unique reads (49.05% mapped). Among 35,462 input transcripts, 21,082 (59.45%) were covered transcripts (Table S1). We identified cleaved targets for miRNAs from the mixed degradome based on CleaveLand 4.0. A total of 528 miRNA-target pairs were identified. Among them, 90 genes were identified to be targeted by 16 DEMs. Here, most of the DEMs and their targets showed similar expression patterns as those in high-throughput sequencing (Figure 4a). The number of target genes corresponding to each DEM varied greatly from 1 (upu-miRn8, upu-miRn7, upu-miR5234) to 24 (upu-miR2592ay) (Table S5), suggesting that upu-miR2592ay might play a complex regulatory role in seed aging.

In order to further analyze the role of target genes, a GO analysis and KEGG pathway analysis were used to obtain functional annotations of these putative 90 target genes of DEMs. In the GO analysis, the ordinate is the GO entry, the left abscissa is the −log_10_ *p* value of the enrichment analysis, and the right abscissa is the gene number. The molecular function was mainly related to “ligase activity”, “nucleic acid binding”, and “inositol hexakisphosphate binding”. Most genes were enriched in the “protein body” in the cellular component. “Protein folding”, “transcription, DNA-templated”, and “response to salt stress” were biological processes associated with seed ageing (Figure 4b). The KEGG results showed that a large number of DEM target genes were annotated into the “spliceosome” pathway. In addition, “protein processing in the endoplasmic reticulum”, ribosome, and “plant hormone signal transduction” also participated in the process of seed aging (Figure 4c).

### 2.5. Validation of miRNA Cleaved Target Genes in Elm Seeds

**Figure 5:**
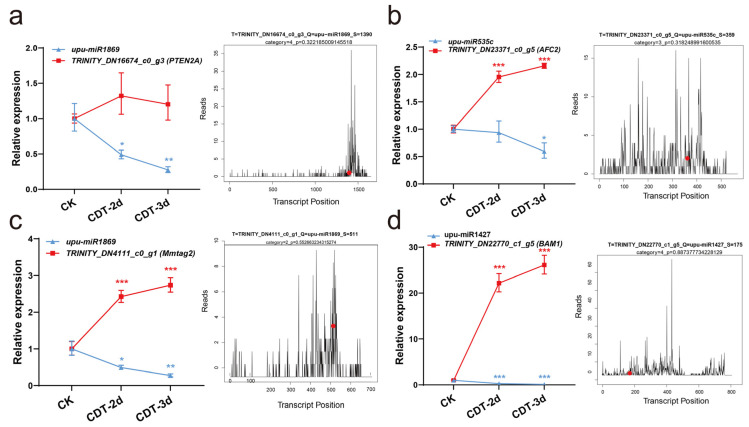
The validation of miRNA cleavage predicted target genes by qRT−PCR. CK, control check; CDT−2d, seeds aged for two days; CDT−3d, seeds aged for three days. (**a**–**d**) MiRNA−target genes confirmed by qRT−PCR (left panel) and target plots (t−plots) of degradome sequencing (right panel). The T−plots diagram shows the full−length distribution of the degradome tags along the target gene sequence. The vertical line with red point indicates the cleavage site of each transcript. Error bars indicate the standard deviation of three replicates. *, *p* < 0.05; **, *p* < 0.01; ***, *p* < 0.001.

**Figure 6:**
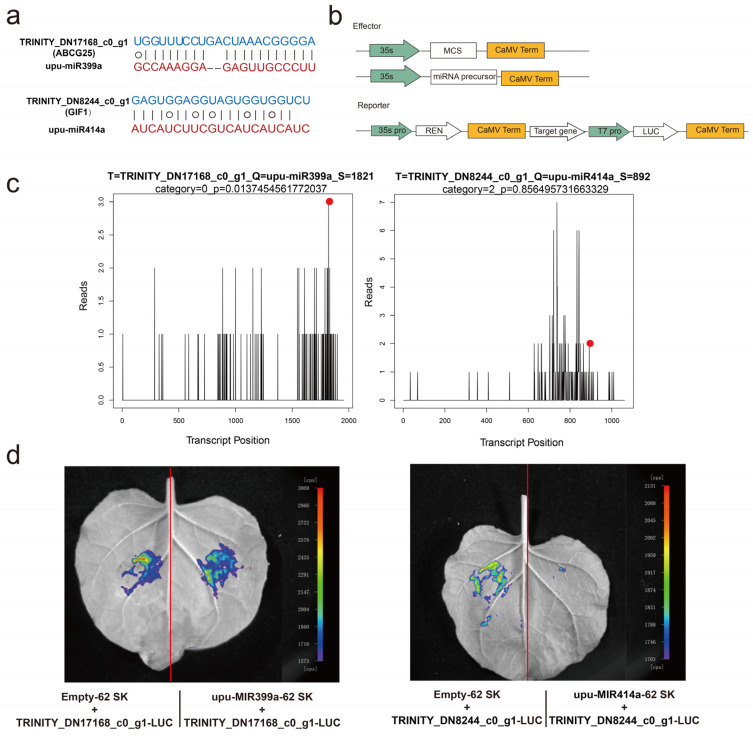
Validation of miRNA cleavage predicted target genes by dual−luciferase assay. (**a**) Sequence match between upu−miR399a, upu−miR414a, and their predicted targets. The target gene (top) and its corresponding miRNA (bottom) are shown in each alignment. The match is displayed in a joint line, and G: U wobble pairing in a circle. (**b**) Construction of the reporter and effector vectors of a dual−luciferase assay. (**c**) Target plots (t−plots) of miRNA targets confirmed by degradome sequencing. The vertical line with a red point indicates the cleavage site of each transcript. (**d**) Dual−luciferase assay of upu−miR399a−ABCG25 (left panel) and upu−miR414a−GIF (right panel). The left side of the red line is the injection area of the control group, and the right side is the injection area of the experimental group. Different colors mean luciferase activity. Cps, signal counts per second.

The transcriptome was used to integrate the expression profiles of miRNAs and their target genes, and to infer the regulation of miRNAs in the aging process of elm seeds. A qRT-PCR was used to detect the expression of four miRNA-target gene pairs (Figure 5a–d, left panel). The corresponding target plots (T-plots) for miRNA-target genes validated by degradome sequencing are shown in the right panel. The degradome data showed that ABC transporter G family member 25 (ABCG25) was the target of upu-miR399a, and GRF1-interacting factor 1 (GIF1) was the target of miR414a (Figure 6a) [21,22]. The T-plot of upu-miR399a-ABCG25 and upu-miR414a-GIF1 showed a single clear peak at the degradation site (Figure 6c). We also used dual-luciferase assays to verify the relationship of upu-miR399a-ABCG25 and upu-miR414a-GIF1 in tobacco leaves. The results of the dual-luciferase assay showed that miRNA specifically negatively regulated their target genes (Figure 6b,d). Above all, the experimental results were consistent with that of degradome sequencing, together indicating that miRNA cleaved the mRNA of target genes and down-regulated their expression.

## 3. Discussion

Since the seed matured, the seed vigor decreased continuously during storage. Our previous study has focused on the physiological and biochemical changes of PCD [4,23], mitochondrial proteome [5], and NO-mediated protein S-nitrosylation [6,7] during the seed vigor decline. In this study, we focused on the gene-level regulation of elm seed aging, analyzed the changes of transcriptome and miRNAs in three aged stages of elm seeds, and described the relationship between these changes during seed aging. Our study attempted to assess the factors affecting the vigor of elm seeds during accelerated aging with integrative omics.

**Figure 7:**
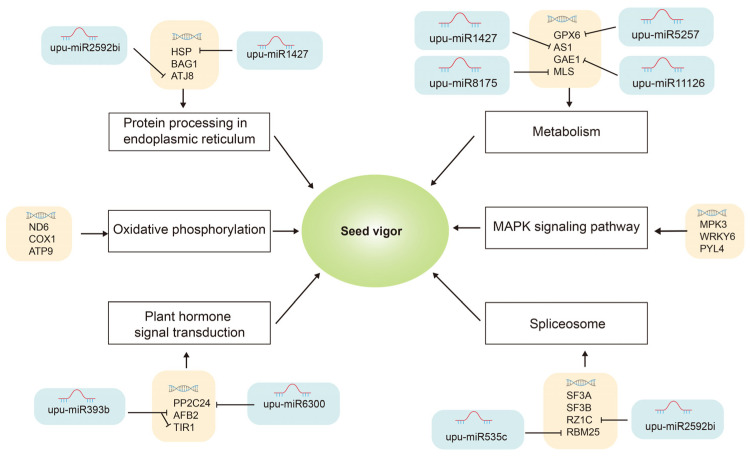
A model for the mRNA and miRNA−mediated mechanism of seed vigor during aging.

In order to better describe the potential relationship between the transcriptome, miRNA-target genes, and seed aging, based on our data, we created a hypothetical network to explain the mechanism of seed aging (Figure 7). At the transcriptional level, *HSP70*, *BAG1* and *ATJ8* (*DnaJ domain containing protein 8*) related to endoplasmic reticulum processing were differentially expressed in seed aging. Upu-miR1427 negatively regulates *HSP*, and *ATJ8* is the target gene of upu-miR2592bi. Metabolism-related miRNAs were differentially expressed during seed aging, and upu-miR1427-AS1 upu-miR5257-GPX6, upu-miR8175-MLS, and upu-miR11126-GAE1 were involved in the regulation of the metabolic pathway. Moreover, upu-miR535c and upu-miR2592bi targeted *SF3A*, *SF3B*, *RZ1C* and *RBM25,* and functioned in the spliceosome. In addition, upu-miR6300 and upu-miR393b were identified to be related to hormone pathways targeting *PP2C24* (*probable protein phosphatase 2C 24*), *AFB2*, and *TIR1*. At the transcriptional level, the expression of *ND6* (*NADH dehydrogenase subunit 6*), *COX1* (*hypothetical protein BevumaM_p019*) and *ATP9* (*ATPase 9*) associated with oxidative phosphorylation was down-regulated. In the MAPK signal pathway, *MPK3* (*Serine/threonine protein kinase 3*), *WRKY6* (*WRKY domain containing protein 6*), and *PLY4* were differentially expressed during seed ageing (Figure 7).

### 3.1. MiRNAs Participate in Regulating Seed Vigor

MiRNAs are critical regulators of gene expression at the post-transcriptional level in plants. They negatively regulate the expression of their target genes, and participate in the physiological processes and the biotic and abiotic stress response of plants. In this study, miRNAs and their targets related to seed aging were identified and analyzed to better understand their roles in seed aging. A total of 111 conservative miRNAs and eight novel miRNAs were identified, among which nineteen conservative miRNAs and three novel miRNAs were differentially expressed during seed aging. Upu-miR398a, upu-miR168, upu-miR390b, and upu-miR393b were up-regulated during seed ageing, while upu-miR11126, upu-miR2592ay, upu-miR5072, and upu-miR3630 were down-regulated (Figure 2b).

Previous studies have reported that miR168a was down-regulated in seeds of rice strain ZR02 after artificial aging treatment. In the rice cultivar Kasalath, the expression of miR168a was positively correlated with seed vigor [16]. In our results, miR168 was up-regulated in aged elm seeds, suggesting that miR168 may have a different regulation mechanism between elm and rice seeds. The miR393-TIR1 regulatory module could manipulate the auxin response. The overexpression of the miR393-resistant mTIR gene delayed the senescence and death rate of *Arabidopsis thaliana* seedlings under salt stress [24]. The expression levels of miR5072, miR3630, and miR2592-y also changed during the process of rice suffering from heavy metal stress, alfalfa under drought stress, or sugarcane internode elongation [25,26,27]. However, the roles of these miRNA in seed aging or related aspects have not been reported, which gives us an idea with regard to the study of their new functions.

### 3.2. Candidate Genes in Aging Elm Seeds

The results of a transcriptome analysis showed that a total of 4900 genes were differentially expressed during seed aging. The number of DEGs increased from 433 in CDT-2d vs. CK to 3248 in CDT-3d vs. CK (Figure 3a), indicating that a large number of genes were involved when the seed vigor decreased from 80 to 50%. These DEGs may largely explain the factors affecting seed vigor.

KEGG enrichment of DEGs showed that the up-regulated genes were mainly involved in the “protein processing in the endoplasmic reticulum” (Figure S1c). Oxidative stress disturbed the protein folding environment of the endoplasmic reticulum cavity, resulting in the accumulation of unfolded and misfolded proteins. This triggers the unfolded protein response (UPR), which can alleviate endoplasmic reticulum stress by up-regulating the expression of the chaperone. During elm seed aging, the expression of some genes, including *HSP70s*, *DNAJs,* and *BAG1* were up-regulated, and they were enriched in protein processing, endocytosis, and the post-translational modification of proteins in the endoplasmic reticulum. Several studies have found that DnaJ alone or together with the heat shock protein 70 (Hsp70) chaperone controls cell homeostasis and participates in protein folding/unfolding, assembly/disassembly, and degradation [28,29,30]. Wang et al. [29] found that SlCDJ2 and Hsp70 might maintain Rubisco activity by stabilizing the level of proteolytic enzymes under heat stress, resulting in the reduced heat-induced damage of Rubisco and improved heat resistance in tomato. In *Arabidopsis thaliana*, BAG1 acts as a cofactor in the proteolytic degradation of misfolded and untransferred plastid proteins in cytosol mediated by Hsc70-4 [31]. These results were consistent with that of An et al. [32] during vigor decline in tobacco seeds. As protein degradation is an inevitable event of cell death during seed ageing, the up-regulation of the HSP70, ATJ8, and BAG1 protein may play a protective role.

Many down-regulated genes were enriched in the oxidative phosphorylation pathway (Figure S1d). Oxidative phosphorylation is the prime energy source of aerobic cells and the main method of ATP production, coupled with an electron transfer chain. Further analysis of the oxidative phosphorylation pathway showed that the expression of genes encoding NADH dehydrogenase, ATPase, NADH-ubiquinone oxidoreductase, and COX oxidase were significantly down-regulated during seed aging (Table S4). It is predicted that the energy metabolism and ATP synthesis ability decreased continuously, which led to energy insufficiency in cells and the aggravation of seed aging. These results align well with previous studies on the mitochondrial proteome of aged elm seeds [5], which suggests that the lower mitochondrial respiration and oxidative phosphorylation after seed aging might be due to the damage to the TCA cycle induced by ROS production.

Genes related to the MAPK signaling pathway was differentially expressed during the aging of elm seeds (Figure S1d). Wang et al. [4] and Li et al. [5] found that the aging process of elm seeds was accompanied by the outbreak of ROS. Studies have shown that ROS is involved in the signal transduction of MAPK [33]. MAPKs participate in the signal transduction of many plant defense responses, responding to pathogens, drought, salt, cold, and ROS stress to regulate plant growth and PCD [34]. In Arabidopsis thaliana, different concentrations of H_2_O_2_ can activate the expression of *AtMPK6* and *AtMPK3* genes [35]. The MAPK-WRKY pathway is necessary for the burst of AVR3a-ETI and INF1-PTIROS in *Nicotiana benthamiana* to be activated by RBOHB [36]. Different MAPKs form a variety of MAPK pathways, modulate cross-talk with various stimulation signals, and form a complex regulatory network. The MAPK pathway related genes *MPK3*, *MPK12*, *RBOHD* (*respiratory burst oxidase homolog protein D*) and *WRKY24* (*WRKY domain containing protein 24*) were up-regulated and *PP2CA* and *WRKY69* (*probable WRKY transcription factor 69*) was down-regulated during the aging of elm seeds (Table S4), which draw our attention to the exact role of the MAPK pathway in ROS-induced PCD during seed ageing.

### 3.3. miRNA-Target Genes Play an Important Role in Seed Aging

In order to better understand the functions of miRNA, the degradome was used to identify their targets. During seed aging, 16,737 target genes were identified for 119 miRNAs, of which nineteen differentially expressed conservative miRNAs and three novel miRNAs target 167 genes (Table S5). According to the degradome, we detected 528 miRNA-target pairs. A total of 90 cleavage sites of 22 DEMs were identified in aged elm seeds (Figure 4a). For example, upu-miR2592ay was predicted to cleave 24 target genes (Table S5), suggesting its function through several pathways in seed aging.

A large number of target genes enriched in metabolism-related genes were predicted as miRNA targets. During seed aging, ROS accumulation is believed to be the cause of seed vigor decline [4,23]. Glutathione S- transferase (GST) catalyzes the sulfhydryl group of GSH to bind to some electrophilic substances, protecting DNA and some proteins from damage. Glutathione peroxidase (GPX) plays an important role in protecting the cell membrane from ROS damage. The Bax protein is an inducer of cell apoptosis. Studies have shown that the expression of tomato GPX in tobacco inhibits cell death induced by salt, heat shock and the Bax protein, thus protecting plant tissues from various stresses [37]. In our data, the continuing decrease of upu-miR5257 during ageing leads to the up-regulation of *GPX6* at CDT-3d. It can be inferred that *GPX6* was activated to fight for the accumulated ROS, but it was too late to rescue the imbalanced redox status and the decreased seed vigor.

With the continuous decline of seed vigor, the genes of the plant hormone transduction pathways have been largely affected. We speculated that miRNA might regulate seed aging through hormone-related pathways. It is generally believed that ABA plays an important role in maintaining seed dormancy. Recent studies have shown that ABA-specific transcription factors ABI3 and ABI5 were involved in the regulation of seed longevity. ABI4 could positively regulate seed vigor by modulating the promoter activity of the gene encoding protein L-isoaspartyl methyltransferase (PIMT) [38] in *Arabidopsis thaliana*. The protein phosphatase PP2C is a key negative regulator of the ABA core signaling pathway [39,40]. In our results, upu-miR6300 was decreased in CDT-2d but increased in CDT-3d (Figure 2b), and its predicted target *At2g29380* (*PP2C24*) has the opposite expression profile (Figure 4a). Combined with the increased mRNA level of ABI4 after ageing (Figure 3c), the ABA signal might be activated to impart seed vigor and longevity during aging.

In *Arabidopsis thaliana*, the auxin signaling activity is related to the acquisition of longevity during seed maturation [41]. In mature seeds, auxin may prolong seed life by destroying the stability of HaIAA27 and increasing the expression of HSFA9 [42]. TIR1/AFBs form co-receptor complexes with Aux/IAAs to facilitate the interaction of ARF with downstream genes [43]. In this study, *TIR* and *AFB* were predicted to be the target genes of upu-miR393b. The continuing increase of upu-miR393b leads to the down-regulation of *TIR1* and a “v” type expression profile of *AFB* during ageing. Thus, it might cause the disorder of the auxin signal during seed aging.

The results of the KEGG pathway enrichment analysis of the target genes showed that splicing pathways were affected by seed aging (Figure 4c and Figure 7). Genes encoding the spliceosome-related components *RZ1C* and *RBM25* were identified as being negatively regulated by miRNAs during seed aging. The splicing of the precursor miRNA or pre-mRNA is a critical step in gene expression regulation after transcription [44]. The negative regulation of splicing factor expression by miRNA may affect the overall splicing events, thus affecting gene expression and participating in the regulation of seed vigor. Our results showed that the interactions between miRNA-target genes not only regulated the mRNA levels of the target genes, but also participated in miRNA regulation through the splicing of the precursor miRNA.

## 4. Materials and Methods

### 4.1. Plant Materials and Seed Treatment

Elm seeds (*Ulmus pumila* L.) were collected from the campus of Beijing Forestry University, Beijing, China. The original germination percentage was 98%, which was tested as described previously [23]. The seeds were stored at −20 °C in tightly closed containers until required for analysis. The CDT was performed as per Hu et al.’s process [23]. The randomly collected seeds were placed in an airtight glass jar (37 °C, 100% relative humidity, balanced for one day). Next, the CDT was initiated until the seed vigor was completely eliminated by the germination test. Seeds treated with CDT for 2 or 3 days were collected and compared with the non-aged seeds (control check, CK). Every treatment was done in triplicate, with at least fifty seeds in each replicate.

For TTC staining, elm seeds were imbibed in ddH_2_O at 25 °C for 12 h. The seed coat was peeled off. The seeds were then divided into two parts and incubated in 0.25% (*w*/*v*) TTC solution at 25 °C in the dark for 12 h. The stained seeds were observed under the stereoscopic microscope and a photo was taken.

### 4.2. RNA Extraction

The seeds of CK, CDT-2d, and CDT-3d were used as samples for subsequent multi-omics analysis. Fifty seeds were placed on the two layers of filter paper in a 9 cm petri dish into which 3 mL of distilled water was added. After four hours of imbibition at 25 °C in the dark, the seed coat was peeled off. The seeds were then cryopreserved in liquid nitrogen and ground into powder in a mortar. The seed powders were used to extract the total RNA.

The total RNA was extracted by Trizol (Invitrogen, Carlsbad, CA, USA) for transcriptome and sRNA library construction. The quality and nucleic acid concentration of all RNA samples were evaluated by agarose gel electrophoresis and a NanoDrop2000C ultra microspectrophotometer, respectively. The degradome sequencing used mixed samples of all RNA. The microRNA, degradome, and transcriptome sequencing data have been deposited in the Sequence Read Archive (SRA) at the National Center for Biotechnology Information (NCBI) under the accession number PRJNA923474.

### 4.3. Transcriptome Sequencing

After the total RNA was qualified, poly-A RNA was purified from 5μg of total RNA using poly-T oligo-attached magnetic beads for two rounds of purification. Following purification, the mRNA was fragmented into small pieces using the buffer, and the cDNA strand was synthesized using mRNA as the template. After purification and buffer elution, the terminal repair was performed, and the poly-A fragments as well as the sequencing joints were connected. The cleaved RNA fragments were then reverse-transcribed to create the final cDNA library in accordance with the protocol for the mRNA-Seq sample preparation kit (Illumina, San Diego, CA, USA). The average insert size for the paired-end libraries was 300 bp (±50 bp). We then performed the paired-end sequencing on an Illumina HiSeq^TM^ 2000 platform following the vendor’s recommended protocol. The sequenced raw data were filtered to remove the adapter, repeated, and low-quality sequencing reads. The sequence quality was then verified using Fast QC (http://www.bioinformatics.babraham.ac.uk/projects/fastqc/ (accessed on 19 April 2023)), including the Q20, Q30 and GC-content of the clean data. Q20 and Q30 indicated the percentage of bases with Phred values >20 and >30, respectively; and GC indicates the ratio of guanine (G) and cytosine (C) of the total base number. Finally, clean data was assembled for subsequent transcriptome analysis based on clean data of high quality. EdgeR software v3.12.1 was used to analyze the differential expression of unigene in each sample, and the false discovery rate (FDR) value of the differential expression was calculated.

To further assess the biological functions of these assembled unigenes, Gene Ontology (GO) (http://geneontology.org/ (accessed on 19 April 2023)) and Kyoto Encyclopedia of Genes and Genomes (KEGG) (http://www.genome.jp/kegg/pathway.html (accessed on 19 April 2023)) enrichment analyses were carried out using DIAMOND [45] with a threshold of E_value < 0.00001. A Venn diagram and expression pattern diagram were drawn using TBtools v1.108 [46], and a bar graph was drawn using GraphPad Prism v8.0.1.

### 4.4. Small RNA Sequencing

The library preparation and sequencing experiments were performed according to standard procedures provided by Illumina Inc. The small RNA sequencing library preparation was performed using the TruSeq Small RNA Sample Prep Kits (Illumina, San Diego, CA, USA). The constructed libraries were sequenced using the Illumina Hiseq2000/2500 platform with a single-end 1 × 50 bp read length. The sequenced raw reads were subjected to the ACGT101-miR (LC Sciences, Houston, TX, USA) program to remove adapter dimers, junk, low complexity, common RNA families (rRNA, tRNA, snRNA and snoRNA), and repeats to obtain clean reads. Subsequently, clean reads with lengths within 18–25 nucleotides (nt) were mapped to specific species precursors in miRBase V22.0 (https://www.mirbase.org/ (accessed on 19 April 2023)) via a BLAST search to identify known miRNAs and novel 3p- and 5p- derived miRNAs. The clean reads mapping to specific species of mature miRNAs in hairpin arms were identified as known miRNAs. The unmapped sequences were blasted against the specific genomes, and the hairpin RNA structures containing sequences were predicated from the flank 120-nt sequences using RNAfold software (http://rna.tbi.univie.ac.at/cgi-bin/RNAWebSuite/RNAfold.cgi (accessed on 19 April 2023)).

The Venn diagram and expression pattern diagram were drawn using TBtools v1.108 [18], and the bar graph was drawn using GraphPad Prism v8.0.1.

### 4.5. Degradome Sequencing and Target Genes Prediction

Mixed mRNA samples of CK, CDT-2d, and CDT-3d were used for degradome sequencing to identify miRNA target transcripts. In the degradome analysis, CleaveLand V4.0 was used to process the obtained raw data for subsequent analysis. The sequences of the sequenced species were aligned to the cDNA database to produce the degradome density file. Split-site prediction software (GSTAr 1.0) [19,20] was used to predict the mRNA sequences of target genes paired with the small RNA of sequenced species. The target gene corresponding to the predicted miRNA and the mRNA in the generated degradation group density file were combinatively analyzed to find out the common mRNA, which was the target gene of the miRNA. The peak classification and score values of the degradation group were given, and the resulting predicted results were plotted (T-plots). The GO (http://geneontology.org/ (accessed on 19 April 2023)) terms and KEGG (http://www.genome.jp/kegg/pathway.html (accessed on 19 April 2023)) pathway of the most abundant miRNA targets were also annotated.

### 4.6. Differential Gene Expression Analysis

There were differences in the expression of the unigene among different samples. Based on the results of express comparison, we used edgeR software v3.12.1 to analyze the differential expression of unigene in each sample, and calculated the FDR value of the differential expression.

The input data of miRNA differential expression analysis were the normalized data (norm value), and the *p*-value calculation model based on normal distribution was used to calculate the *p*-value. The chi-square (2 × 2) test was used to analyze the difference between the two groups of samples.

DEGs and DEMs were selected with foldchange > 2 or foldchange < 0.5 (|log_2_FC| ≥ 1), with the statistical significance (FDR or *p* value < 0.05) as the threshold. The foldchange represents the comparison of group difference multiples.

### 4.7. qRT-PCR Analysis

Total RNA was extracted from samples using an RNA Isolation Kit (Aidlab, Beijing, China) and then reverse transcribed into cDNA using a TRUEscript RT MasterMix (for real-time PCR) (Aidlab, Beijing, China). A CFX96 qRT-PCR platform (BioRad, Hercules, USA) was used for the qRT-PCR analysis. The actin of elm was used as the internal reference. Each reaction system contained 2.0 μL of diluted cDNA, 1.4 μL of forward primer (10 μM), 1.4 μL of reverse primer (10 μM), 10 μL of 2× SYBR Premix Ex Taq fluorescent reagent (QIAGEN, Duesseldorf, Germany), and 5.2 μL of ddH_2_O. The qRT-PCR reaction conditions were as follows: 95 °C for 2 min, 40 cycles at 95 °C for 5 s, and 60 °C for 10 s. All reactions were performed in triplicate, and the average and standard deviation were calculated. The primers for qRT-PCR are listed in Table S6.

MiRNAs were isolated from samples according to the instructions of an EASYspin plant miRNA isolation kit (Aidlab, Beijing, China). Based on the poly (A) tailing principle, the miRNAs were reversely transcribed into cDNA with a miRNA cDNA first-strand synthesis kit (TIANGEN, Beijing, China). The 5.8S rRNA of elm was used as the internal reference. A q RT-PCR was then performed in a reaction system of 2.0 μL of diluted cDNA, 0.4 μL (10 μM) of forward primer (10 μM), 0.4 μL of reverse primer (10 μM), 10 μL of 2 × SYBR Green Mix (TIANGEN, Beijing, China), and 7.2 μL of ddH_2_O. The reactions were carried out in triplicate on a CFX96 qRT-PCR system under the following amplification conditions: denaturation at 95 °C for 15 min, followed by 40 cycles at 94 °C for 20 s and 60 °C for 34 s. qRT-PCR forward primers were designed for the miRNAs and are listed in Table S6.

### 4.8. Dual-Luciferase Assay

The dual-luciferase transient expression system was used to study the regulation of miRNA on the target genes [47]. We used the experimental method of Wang et al. [48], with modifications. The full-length sequences of pre-microRNAs were constructed into the pGreen II 62-SK vector. The target sequences of microRNAs of 200–400 bp were constructed into the pGreen II 0800-LUC vector. Both of the plasmids were transferred into *Agrobacterium tumefaciens* (GV3101, pSoup-p19, Weidibio, Shanghai, China). The *A. tumefaciens* strains were cultured and then dissolved with an infiltration medium (10 mM 4-morpholine ethane sulfonic acid, pH 5.6, 10 mM MgCl_2_ and 150 mM Acetosyringone) to OD_600_ = 0.8. For dual-luciferase assay, the volume ratio of *A. tumefaciens* mixtures of SK and LUC was 2: 1. The prepared mixtures were then injected into the leaves of *Nicotiana benthamiana*. Three days after injection, the activity of firefly luciferase and Renilla luciferase were detected in D-Luciferin potassium salt (15 mg/mL, BioDee, Beijing, China) by Night Shade LB 983 (Berthold Technologies, Bad Wildbad, Germany). Dual-luciferase assays were performed with five biological replicates, and the primers used for the SK and LUC vectors are listed in Table S6.

### 4.9. Statistical Analysis

Gene relative expression levels were calculated using the 2^−∆∆CT^ method with three biological replicates. GraphPad Prism v8.0.1 (GraphPad Software, San Diego, CA, USA) was used for data processing and statistical analysis. The statistical significance was calculated using a two-tailed Student’s *t*-test. Error bars indicate standard error (SE). Values of *p* < 0.05 were considered statistically significant. The statistical methods for omics data were described in Section 4.6.

## 5. Conclusions

To the best of our knowledge, there were more studies that focused on the function of miRNA in seed development/germination/dormancy, and less that focused on seed vigor/aging/longevity. In addition, less attention was paid to forest seeds compared to crop seeds. In our study, the network of mRNA, miRNA, miRNA-target genes in the aging process of elm seeds was outlined. A total of 119 miRNAs were identified, including 111 conservative miRNAs and eight novel miRNAs. A total of 4900 genes and 22 miRNAs were differentially expressed during the aging of elm seeds. Furthermore, 528 miRNA-mRNA pairs were identified, and the negative regulation of several miRNAs on their target genes was verified by experimental approaches. The integrated analysis of the miRNAome, transcriptome, and degradome in this study provides new insights into the regulation network of elm seed aging, and provides much information for further studies on the roles of candidate miRNAs and genes related to seed vigor.

## Data Availability

The data is contained within the manuscript and Supplementary Materials.

## Supplementary Materials

The following supporting information can be downloaded at https://www.mdpi.com/article/10.3390/plants12081719/s1, Figure S1: Analysis of transcriptome data; Table S1: Summary of illumina sequencing for aging elm seeds; Table S2: Statistics of small RNA libraries analyzed by high-throughput sequencing; Table S3: Statistics of identified novel miRNAs; TableS4: Comparison of differentially expressed genes (DEGs) in different groups; Table S5: Target genes predicted of conservative miRNA and novel miRNA by GSTAr v1.0 and degradome sequencing; Table S6: Primer sequences.
