# Supplementary material for: Integrated Analysis of miRNAome and Transcriptome Identify Regulators of Elm Seed Aging
Source: Plants (Basel). 2023 Apr 20;12(8):1719. doi: 10.3390/plants12081719 (PMC10140922; doi:10.3390/plants12081719)
Supplement: Supplementary file 1 [file plants-12-01719-s001.zip › Supplementary Figures.pdf]

plants

Supplementary Materials

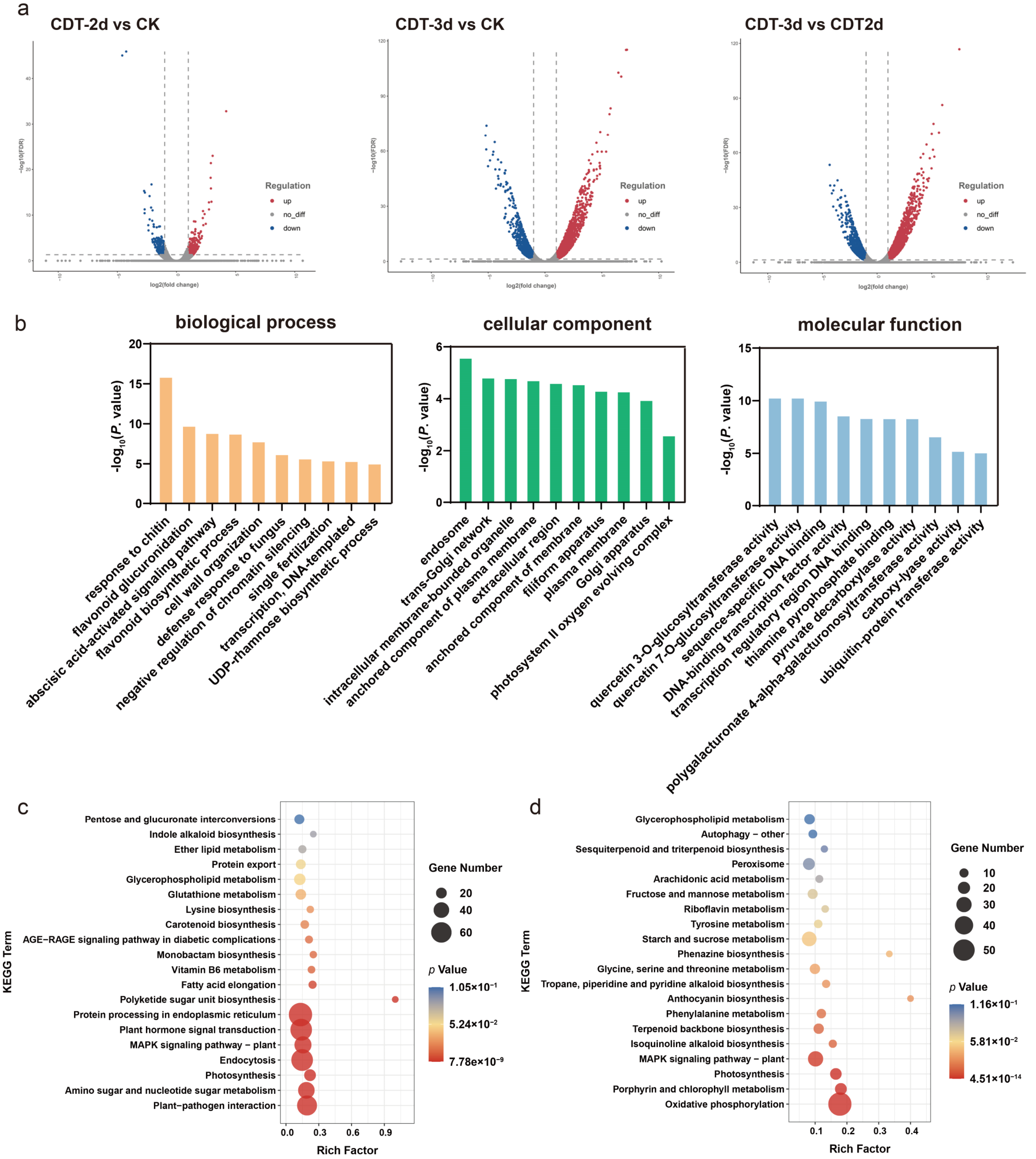

Figure S1. Analysis of transcriptome data; Table S1**:** Summary of illumina sequencing for aging elm seeds.
